# Experiences of the Management of Uncertainty Amongst Musculoskeletal First Contact Practitioners Working in Primary Care

**DOI:** 10.1002/msc.70062

**Published:** 2025-02-07

**Authors:** Matt Parselle, Sue May

**Affiliations:** ^1^ MSK Physiotherapy Royal Devon University Healthcare NHS Foundation Trust Exeter UK; ^2^ School of Health Professions Faculty of Health University of Plymouth Plymouth UK

**Keywords:** first contact practitioner, musculoskeletal, physiotherapy, primary care, uncertainty

## Abstract

**Aim:**

To develop a deeper understanding of strategies used to manage uncertainty by Musculoskeletal First Contact Practitioners (MSK FCPs), including barriers to and facilitators for these strategies.

**Background:**

MSK FCP services provide patients with an alternative to seeing their GP regarding MSK complaints. Research suggests that the role demands different skills and attributes from traditional physiotherapy roles, including the ability to deal with greater clinical uncertainty. There is a lack of research evaluating the strategies FCPs find most helpful for managing uncertainty.

**Method:**

A qualitative study using semi‐structured online interviews. Participants were recruited using convenience sampling. Data was analysed using Braun & Clarke's reflexive approach to thematic analysis. The research was underpinned by a theoretical framework of hermeneutic phenomenology.

**Findings:**

Nine participants were recruited. Three main themes were developed: (1) Being comfortable with being uncomfortable; (2) Teamwork makes the dream work and (3) Navigating uncertainty with patients.

**Conclusion:**

This study provides further insight into how FCPs manage uncertainty. Management of uncertainty was influenced by many factors, including: clinician experience, patient complexity and wider medical knowledge, fear of over‐medicalising patients, communication and consultation styles and having protected non‐clinical time. Recommendations for clinical practice include: consideration of the challenges facing FCPs, and what support is needed to maintain staff retention, health and wellbeing; consideration of how FCPs might best approach meeting the needs of an ageing population and supporting change in health and wellness behaviour. The key to successful management of uncertainty was having a supportive team which encouraged open non‐judgemental discussions about uncertainty.

## 1 Introduction

### 1.1 Background

Musculoskeletal First Contact Practitioners (MSK FCPs) are physiotherapists with advanced clinical skills that provide expert assessment, diagnosis and management plans for patients with MSK disorders without the need for a prior GP appointment (Chartered Society of Physiotherapy 2021). MSK FCPs will frequently see complex patients with multiple co‐morbidities and signs or symptoms which can appear vague and/or be masqueraders for non‐MSK pathologies. A key requirement of the role, therefore, is the ability to manage uncertainty as it relates to, for example, diagnosis, prognosis, referral, management, communication and boundaries of the role (Mercer and Hensman‐Crook 2022).

The MSK FCP role has emerged in recent years in response to growing pressures on GPs (Baird et al. 2016). In 2019, the Additional Roles Reimbursement Scheme (ARRS) was introduced in England to improve patient access to a greater diversity of multi‐disciplinary teams in General Practice, including FCPs (NHSEI 2019).

The research indicates that FCP‐led models in General Practice provide safe, effective and cost‐beneficial management of patients with MSK disorders when compared with traditional GP‐led consultation models (Walsh et al. 2024).

However, there have been concerns raised with how physiotherapists manage and respond to the increased levels of uncertainty related to this relatively new role (Goodwin et al. 2021; Greenhalgh, Selfe, and Yeowell 2020; Ingram, Stenner, and May 2023; Langridge 2019), with intolerance of uncertainty being linked to work‐related stress and burnout among FCPs (Ingram, Stenner, and May 2023).

Uncertainty tolerance in healthcare has been defined as: ‘the set of negative and positive psychological responses—cognitive, emotional, and behavioural—provoked by the conscious awareness of ignorance about particular aspects of the world’ (Hillen et al. 2017, 70).

Responses to uncertainty vary widely. In some, it may provoke fear, stress and a feeling of professional failure, and therefore may be viewed as something to be mitigated or avoided. Others may be attracted to uncertainty and enjoy the ambiguity and complexity that it entails (Hillen et al. 2017). Negative responses to uncertainty may lead to clinicians seeking dysfunctional ways out, such as an over‐reliance on tests and investigations, or getting others to deal with the problem via poorly focussed referrals or passing patients on to colleagues. This in turn can impact the safety and quality of patient care, and lead to clinician burnout (Danczak and Lea 2017a; Pomare et al. 2018).

Uncertainty in healthcare pertains to numerous unknowns; however, it is thought to be particularly prevalent in primary care, largely due to the undifferentiated nature of many presentations, and patients seeking care at an early stage when a diagnosis is not clear cut. This requires a different approach to clinical reasoning and decision‐making that FCPs may be unaccustomed to (Atkinson, Ajjawi, and Cooling 2011).

### 1.2 Current Literature

**FIGURE 1:**
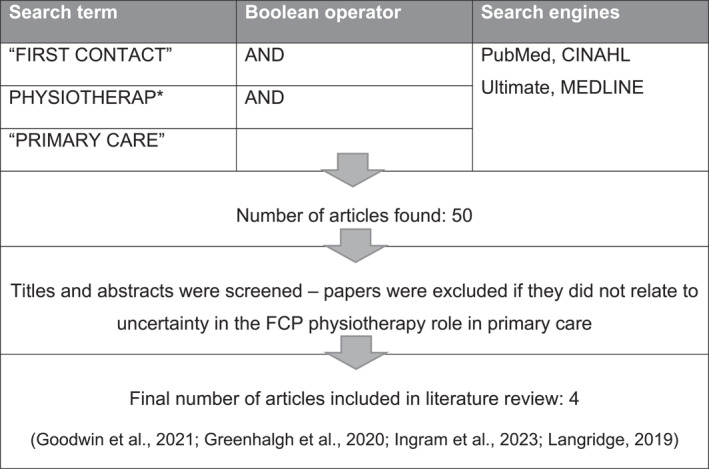
Literature search strategy.

The literature search strategy is summarised in Figure 1.

Analysis of the included papers was guided by the use of the COREQ criteria (Tong, Sainsbury, and Craig 2007). A worked example is included in Appendix 1.

Currently, there is little research investigating how physiotherapists adapt to working in primary care.

All papers identified in the literature search followed a qualitative semi‐structured interview approach (Goodwin et al. 2021; Greenhalgh, Selfe, and Yeowell 2020; Ingram, Stenner, and May 2023; Langridge 2019).

Langridge (2019) highlighted that the FCP role requires a deeper level of understanding and analysis than a traditional physiotherapy role, stressing the importance of understanding the influences on FCPs' clinical reasoning and decision‐making processes.

All four papers indicated concerns around the additional burden of responsibility felt by FCPs due to uncertainty related to the diagnosis and management of unscreened patients. This included: fear of missing serious pathology, differentiating between MSK and non‐MSK conditions, and the complexity of patients who often have multiple co‐morbidities.

FCP participants in the papers by Goodwin et al. (2021), Greenhalgh, Selfe, and Yeowell (2020), and Ingram, Stenner, and May (2023) also discussed uncertainty as it related to the lack of understanding of the role (from patients and members of the primary care team), and the impact of uncertainty on FCPs' own wellbeing, including feelings of isolation, stress, and burnout, leading them to consider leaving the role.

Unlike Langridge (2019), these papers discussed the management of uncertainty, including the use of safety netting and having direct access to GPs. However, this was not their primary focus. Goodwin et al. (2021) only touched on the subject of uncertainty very briefly in relation to the unpredictable nature of the work and the use of safety netting to mitigate this. Greenhalgh, Selfe, and Yeowell (2020) and Ingram, Stenner, and May (2023) focused on more general experiences of uncertainty rather than its management. Participants in Greenhalgh, Selfe, and Yeowell (2020) were based in a single FCP service, which may have reduced the transferability of the findings.

In the case of Langridge (2019) and Greenhalgh, Selfe, and Yeowell (2020), the author/interviewer was working in the same environment as the participants, which could have influenced data collection and analysis. Attempts were made to mitigate this—for example, the data in Langridge (2019) was coded by an independent researcher. In the study presented here, further attempts were made to mitigate the researcher's influence by including participants not working in the same environment as the researcher.

### 1.3 Aims and Objectives

It appears that uncertainty is a key aspect of the FCP role. However, the research has only explored *management* of uncertainty to a very limited extent. The aim of this study is to develop a deeper understanding of what strategies, skills and traits FCPs find helpful for managing uncertainty, as well as the barriers to and facilitators of these strategies. It is hoped that this will provide the basis for further research in this area, with the aim of reducing clinician burnout, increasing staff retention, and ultimately improving patient outcomes. It is also hoped that it will aid the development of training and support to help FCPs manage uncertainty.

The objectives will be to: (1) Explore what strategies, skills and traits FCPs use to manage uncertainty and (2) Identify barriers to/facilitators for the management of uncertainty.

## 2 Methodology

### 2.1 Research Approach

A qualitative study using in‐depth semi‐structured online interviews was conducted in accordance with the COREQ criteria (Tong, Sainsbury, and Craig 2007).

Semi‐structured interviews offered some structure to the process, enabling the research aim to be explored in depth, while at the same time allowing the flexibility to investigate unexpected responses. This was preferred to the structured interview, which follows a more rigid questionnaire format and may not have offered the same depth, and the unstructured interview, which may have allowed too broad an area to explore (Petty, Thomson, and Stew 2012).

While the online interview format could have adversely affected researcher‐participant rapport, it was chosen as it removes geographical constraints, and is considered a preferred method for participants (Archibald et al. 2019).

### 2.2 Theoretical Framework and Qualitative Design

A qualitative design was chosen over a quantitative approach, so as to address the study aim and objectives by gaining deep insight into the ways that research participants perceive, interpret and explain their experiences (Stenner, Mitchell, and Palmer 2017). This choice also brought the study in line with previous research in this field.

The study was underpinned by hermeneutic phenomenology. This approach aims to interpret lived experience and unearth meaning through understanding and interpretation of data, and was therefore considered appropriate to address the research aim (Laverty 2003).

### 2.3 Reflexivity and Researcher Characteristics

It is acknowledged that the position of a researcher can have a significant impact on the research process. Being reflexive of personal circumstances, bias, opinions and experience is therefore vital (Freshwater 2005). The lead author of this study is a novice in the field of research but is an experienced MSK clinician and FCP, who has considerable experience discussing clinical matters with other FCPs, and who believes that the development of the FCP role is a positive one.

Using reflexivity, the researcher acknowledged their own preunderstanding to enhance the trustworthiness of the research process and used their insider perspective to build rapport with the participants and gain richer data. To further enhance study trustworthiness, the researcher interrogated their impact through regular discussions with the project supervisor, and through use of field notes including the researcher's reflections following each interview (Braun and Clarke 2022). There was also regular discussion between the researcher and supervisor during coding and theme creation.

### 2.4 Ethics

Ethical approval for this study was obtained through a University Faculty of Health Research Ethics and Integrity Committee (Project ID: 4892; Date: 12 March 2024). All participants provided verbal informed consent prior to the interview. All data were stored in accordance with the University's data management policy.

### 2.5 Pilot Interview

A pilot interview was recorded with a colleague of the researcher to help refine the language, structure and flow of questions in the interview guide. The final guide is shown in Appendix 2.

### 2.6 Participant Selection and Data Collection

Participant selection criteria are listed in Table 1.

**TABLE 1:** Selection criteria.

Inclusion criteria	Exclusion criteria
MSK FCPs currently working in primary care in England^a^ (may include those working in a split role, e.g., FCP & APP/ESP)	Anyone who does not meet the inclusion criteria
	Not able to speak English

^a^ Only FCPs working in England were eligible as Scotland, Wales and Northern Ireland are not governed by the capability roadmap for England (Health Education England 2021).

**FIGURE 2:**
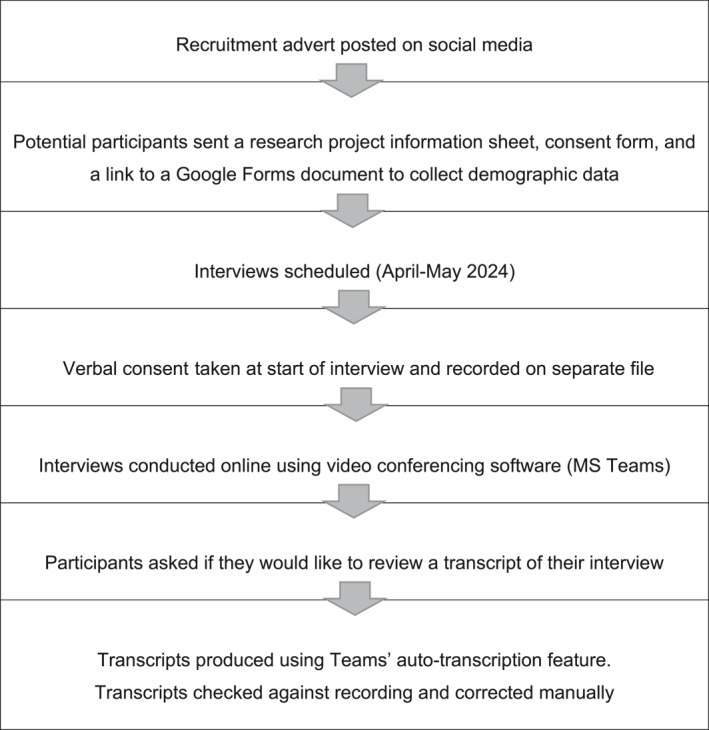
Recruitment/data collection process.

Participants were recruited using convenience sampling. The recruitment and data collection process is illustrated in Figure 2.

It was acknowledged that there is disagreement regarding sample size and when to stop data collection because of the differences of opinion around the traditional idea of ‘data saturation’ (Braun and Clarke 2019; Varpio et al. 2016). Instead, the idea of theoretical sufficiency was used. Theoretical sufficiency places emphasis on how meaningful the data appears. If it is felt that the data adds no new perspectives and understandings to the research subject, it is reasonable to conclude that an appropriate sample size has been achieved (Malterud, Siersma, and Guassora 2016; Birt et al. 2016).

### 2.7 Data Analysis

Reflexive thematic analysis (TA) was chosen as the method of data analysis. TA is a theoretically flexible interpretive tool that can be used to provide a rich understanding of patterns (themes) within interviews and across data (Braun and Clarke 2006). This was of relevance to the current study, and considered important if the study findings were to have clinical applicability (Braun and Clarke 2021). TA was considered particularly useful for addressing the research aim as it captures the deep meaning of participants' comments that can be lost with a more word‐based analysis (Guest, MacQueen, and Namey 2012).

This study adopted an experiential approach to TA in aiming to capture and explore the participants' perspectives and understandings of the management of uncertainty (Braun and Clarke 2022).

The data analysis took a largely inductive approach, accepting that codes and themes can change or be added/removed during analysis. This enabled the researcher to derive meaning and develop themes away from preconceived ideas and be reflexive in this process.

The project formed part of the researcher's MSc qualification; therefore, data analysis was carried out by the researcher with support from the project supervisor. Themes were refined through discussions between the researcher and supervisor, and during the writing‐up process.

The TA process was guided by the six phases as outlined by Braun and Clarke (2006) (see Table 2).

**TABLE 2:** Phases of thematic analysis.

Phase	Description of process
1. Familiarising yourself with your data	Transcribing data (if necessary), reading and re‐reading the data, noting down initial ideas
2. Generating initial codes	Coding interesting features of the data in a systematic fashion across the entire data set, collating data relevant to each code
3. Searching for themes	Collating codes into potential themes, gathering all data relevant to each potential theme
4. Reviewing themes	Checking if the themes work in relation to the coded extracts (Level 1) and the entire data set (Level 2), generating a thematic ‘map’ of the analysis
5. Defining and naming themes	Ongoing analysis to refine the specifics of each theme, and the overall story the analysis tells, generating clear definitions and names for each theme
6. Producing the report	The final opportunity for analysis. Selection of vivid, compelling extract examples, final analysis of selected extracts, relating back of the analysis to the research question and literature, producing a scholarly report of the analysis

## 3 Results

Interviews with nine participants were conducted. The demographic data of participants are presented in Table 3. The average interview duration was 52 min (range 38–68 min). Only one participant worked for the same employer as the researcher; however, they did not work directly together.

**TABLE 3:** Demographic data.

Participant ID	Gender	Ethnicity	NHS pay band/equivalent	Years qualified as physio	Years as FCP	Employer	Split/portfolio role?	Region of England
FCP 1	Male	White	7	4	2.5	NHS	Yes	South West
FCP 2	Male	White	7	14	2.5	Private/other	No	North West
FCP 3	Female	White	8a	22	3.5	NHS	No	East
FCP 4	Female	White	8a	34	5	NHS	Yes	South West
FCP 5	Male	White	8a	20	6	Other	No	North West
FCP 6	Female	Asian	7	19	3	PCN	No	East Midlands
FCP 7	Female	White	7	16	0 years 10 months	NHS	Yes	South West
FCP 8	Male	White	7	14	3	PCN	No	South West
FCP 9	Female	White	8a	30	7	NHS	Yes	South West

Two participants requested to check their interview transcripts. No alterations to the transcripts were made.

In general, participants considered uncertainty to be pervasive in primary care and that the management of uncertainty should be seen as a fundamental part of the FCP role.

Uncertainty was seen in a positive and negative light, as illustrated by the two quotes below:

I find the uncertainty interesting and stimulating. I think I'd get bored if I wasn't [uncertain]. I like thinking about things, I like variety.

(FCP 8)

[Management of uncertainty] can be stressful… in my last role [there were] days where I thought I can't be bothered to go in today or this is becoming quite tedious… burnout, I guess, and low job satisfaction… I still get days where I'm querying whether or not I want [to pursue] physio forever.

(FCP 1)

## 4 Themes

Three main themes were developed from the data (see Table 4). Further details, including theme synopses and related codes, are given in Appendix 3.

**TABLE 4:** Themes and subthemes.

Theme	Subtheme
1. Being comfortable with being uncomfortable	Barriers & facilitators to being comfortable with uncertainty
2. Team work makes the dream work	Making uncertainty normal
3. Navigating uncertainty with patients	—

### 4.1 Theme 1: Being Comfortable With Being Uncomfortable

Participants alluded to the recognition of when they felt uncertain as being the first stage of managing uncertainty.

The first thing for me is… recognising when I'm uncertain. And being comfortable [laughs], or as comfortable as one is, with acknowledging that and recognising when it doesn't quite feel right. And it might be in a session, or it might be afterwards, I think, ‘Hmm, I'm not sure’…

(FCP 8)

Being comfortable with uncertainty was often linked to clinical experience. There was concern that less experienced FCPs may struggle in the role.

The juniors coming up from Band 6… they're good… but they've got no clinical experience… this role requires you to know what's not normal, and unless you've seen a lot of normal, you can't… a lot of [the uncertainty] is mitigated by the fact that I am really experienced.

(FCP 4)

Some participants felt that experience was a ‘double‐edged sword’: although it may facilitate the management of uncertainty, it also made them realise how much they did not know.

Starting in the role, I was much more uncertain than I am now… I manage it a lot better and I can see that that has a positive effect on the patients. But the more you do the role, the more you realise there's so many things you don't understand because you're delving more into the medical side of things because you're seeing that person at first contact.

(FCP 3)

### 4.2 Sub Theme: Barriers & Facilitators to Being Comfortable With Uncertainty

Uncertainty was commonly linked to clinical complexity, diagnosis, differentiating between MSK and non‐MSK presentations, and the fear of missing serious pathology, with participants identifying a number of barriers and facilitators to the management of these areas.

#### 4.2.1 Wider Medical Knowledge

Participants placed emphasis on the need for a wider medical knowledge outside their more familiar MSK knowledge base. This appeared to instil more confidence in them when discussing differential diagnoses with patients.

It's important to be confident with identifying whether there might be a medical cause for [the patient’s symptoms]… Having an awareness of things that might masquerade as the symptoms and having the knowledge to say to that patient, currently you're not experiencing any of those symptoms, reassuring them, and hopefully settling their concerns.

(FCP 1)

Training, sometimes delivered by other members of the primary care team, was seen as helpful in expanding medical knowledge and managing uncertainty.

Our training is fantastic. I cannot fault my Trust… it's done on a very regular basis. We get doctors in, nurses, paramedics… so that mitigates some of the risk.

(FCP 4)

However, some participants highlighted a lack of training.

[Management of uncertainty is] something that hasn't been taught. It's something that's self‐taught.

(FCP 1)

#### 4.2.2 Use of Investigations

Many participants used investigations such as imaging or blood tests to help mitigate uncertainty. However, there was recognition that such investigations were not always conclusive.

Investigations can reassure you, but they can sometimes falsely reassure you. You can't say that they haven't got, say, malignancy, it's not going to present on an X‐ray unless it's quite advanced.

(FCP 3)

There were concerns that uncertainty would lead to more investigations with the potential of over‐medicalising the patient.

Faced with uncertainty, we run the risk of performing additional tests, whether that's bloods, whether that's imaging… that can be costly from a healthcare perspective, it can be time consuming. It runs the risk of over‐medicalising the patient as well.

(FCP 1)

Participants mitigated the risk of increasing patient anxiety by pre‐emptively contextualising potential imaging results as ‘normal’.

Sometimes I feel forced to order something because the patient's anxious. I might prep them [by saying], ‘This is likely to show some age‐related changes which [are] quite normal for the general population’… [so that] it doesn't make them even more anxious, and that usually works.

(FCP 3)

#### 4.2.3 Screening for Serious Pathology

Participants frequently worried about ‘missing’ something serious, particularly with complex presentations. When screening for serious pathology, participants often used a clinical reasoning model based on their level of concern. However, uncertainty could make them risk‐averse and lead to yet more investigations.

…the most important thing initially is to make sure you're not dealing with something sinister… It comes down to your index of suspicion… how concerned are you about that patient… if there is a lot of uncertainty, you have to plough forward with investigations.

(FCP 3)

Participants often relied on subjective history taking and red flag questions as well as physical examination findings to mitigate the risk of missing serious pathology. However, there was acceptance that this strategy was not always reliable, and instead they relied on ‘gut instinct’.

[The patient] had no other red flags, but you just don't know. Did he have carpal tunnel? Did he have a rheumatology problem? Is this cancer related… because he's got this sudden onset of neurological symptoms? You have to trust your gut.

(FCP 4)

Safety netting was seen as essential; however, it needed to be specific around time frames and signs or symptoms to look for. Clinicians were mindful of not scaring patients unnecessarily, while also concerned that patients might not take their advice seriously.

You can tell them to go to A&E, but [patients] don't want to spend eight hours in A&E… they don't get the urgency even with cauda equina symptoms, because the minute you [say] it's a very rare thing to not scaremonger them, it also takes that urgency out of it.

(FCP 4)

#### 4.2.4 Time Management

Time management was seen as a significant barrier to the management of uncertainty due to the impact it had on clinical reasoning and cognitive processes.

We have 30‐minute appointments… if you've got a barn‐door MSK patient with a straightforward single problem, then 30 minutes seems like a luxury. But when you've got somebody more complex, that time could influence your sense of confidence and competence.

(FCP 5)

A lack of understanding of the FCP role from primary care colleagues was occasionally cited as a barrier to time management, with FCP clinics being used as ‘dumping grounds’ for the more complex patients on a GP's caseload.

Having flexibility and control over clinics was seen as a facilitator to the management of complex patients, allowing more time for clinical reasoning and reducing stress.

[It’s] not the nature of our role to get [patients] back in as follow‐ups, but we do… if [the FCP is] not sure and it makes [them] feel better.

(FCP 4)

### 4.3 Theme 2: Team Work Makes the Dream Work

Participants stressed the importance of having an approachable and supportive team when it came to effective management of uncertainty. Ease of access to GPs, for example, was seen as paramount.

Having an open‐door policy and having a friendly GP on the other side of that door rather than a GP that's flustered himself… [The discussion is] often brief and to the point, but that doesn't matter.

(FCP 4)

This importance of good working relationships extended to the wider primary care team and colleagues in secondary care.

It's all about team working and knowing your own scope of practice and picking the brains of not just doctors, but pharmacists or nurses [too].

(FCP 9)

Because we're employed by secondary care, we've got good links with the hospitals, so I can speak to a specific consultant… [and because] they know me… they're very responsive.

(FCP 3)

Participants indicated that a barrier to their management of uncertainty was *lack* of access to GPs due to, for example, not working on the same site or days as the doctors, as well as the perception that GPs were too busy to be ‘pestered’.

Having complex patients… makes you appreciate a close‐knit MDT. When you're not on site, it’s harder than being able to have a face‐to‐face conversation with the GP.

(FCP 3)

The use of IT systems—such as EMIS and Advice & Guidance—to communicate with primary and secondary care teams was seen as a useful facilitator, with participants being aware of not wanting to waste GP time with less urgent enquiries.

### 4.4 Sub Theme: Making Uncertainty Normal

Participants valued having a supportive MSK team which encouraged open discussions about uncertainty in a perceived ‘safe space’.

It's OK for [uncertainty] to be an issue. You don't have to feel you’re at fault if you don't have the answers to every patient that walks through the door… and if you dismiss that, you'd probably be working unsafely. [An] open culture where [uncertainty is] part of the working week is important.

(FCP 9)

Having direct access to, and discussions with, MSK colleagues was seen as critical. Participants made particular reference to the value of protected non‐clinical time for complex case discussion, supervision and training to improve their confidence in managing uncertainty and any related stress.

Conversely, *lack* of support from colleagues and protected non‐clinical time was seen as a barrier to managing uncertainty, and linked with feelings of isolation, leading to burnout.

[Sometimes] I feel like we are there to just take the edge off the numbers that are coming through the surgery. There's little time [for] clinical discussions or supervision, training, development… That's made me sceptical about the role, [my employers not offering]… support.

(FCP 1)

### 4.5 Theme 3: Navigating Uncertainty With Patients

Participants generally felt that having honest and open discussions with patients about uncertainty was a positive strategy, and was usually well received.

I'm comfortable sharing my uncertainty, and I deliberately do it when appropriate… It's important to be honest with people to move them away from a mentality that I can ‘fix’ them.

(FCP 8)

However, the success of such discussions could vary depending on the patient.

Relaying uncertainty can be challenging if you've got people that are distressed, or [have] challenging expectations, wanting immediate answers… That's when it can be difficult, and my perception is that they've lost confidence in me.

(FCP 1)

#### 4.5.1 Consultation Style

Participants found consultation style a facilitator in navigating uncertainty with patients, with the ICE (Ideas, Concerns, Expectations) model (Pendleton et al. 2003) being frequently cited, as well as a need for flexibility.

I'm careful to ask people what they think's going on and what they're expecting… There's usually other stuff – lifestyle, worries – that you have to explore, otherwise you're at risk of just getting them out of the room, but you haven't made it better for the health system, let alone the patient.

(FCP 8)

My communication style is tailored to the patient, to their knowledge, their anxiety levels, their personality…we need to be quite chameleon like.

(FCP 9)

#### 4.5.2 Shared Decision‐Making

Most participants saw shared decision‐making as a facilitator in navigating uncertainty with patients.

Being honest with patients pays dividends because we're having an honest conversation about what they think, what I think… and that leaves us in a more equal place… it's a better approach than thinking that *I've* got to know it all. We're two human beings trying to work out the best course of action.

(FCP 8)

However, there was acknowledgement that giving patients a choice could have a negative impact on the clinician‐patient relationship.

Patients don't always want options ‐ they want a solution… [the patient will] say, ‘How do I know, you're the [one] with the qualifications… you tell me what to do’… It can backfire.

(FCP 5)

## 5 Discussion

This research adds to the small body of FCP literature investigating uncertainty in primary care. There were a number of themes that echoed the findings of previous research, including uncertainty as it relates to patient complexity, diagnosis, and the fear of missing serious pathology, as well as the impact of uncertainty on the health, wellbeing and motivation of FCPs.

However, this study is unique in that it provides further insight into how FCPs manage uncertainty, and the barriers and facilitators involved.

There was acknowledgement that uncertainty was part of the job and could not, or should not, be avoided. This correlates with the existing primary care literature, which suggests that uncertainty should be embraced and that maladaptive responses to uncertainty have detrimental effects on clinicians and patients (Danczak and Lea 2017b; Mishel 1988; Simpkin and Schwartzstein 2016).

FCPs frequently found the management of uncertainty challenging and, in some cases, exhausting and stressful, leading to burnout and reduction of working hours or consideration of leaving the role altogether. However, uncertainty was also seen in a positive light, making the role exciting and stimulating, and encouraging FCPs to broaden and improve their knowledge and skills. This reflects the large volume of research from numerous other disciplines, within and outside of healthcare, demonstrating a wide variation in responses to uncertainty both between individuals and within the same individual depending on the context (Hillen et al. 2017).

### 5.1 Communication

The key factor underpinning this study, and one which impacted a number of the themes and sub themes was communication.

The importance of good communication and direct access to colleagues has been noted in previous FCP research (Goodwin et al. 2021; Greenhalgh, Selfe, and Yeowell 2020; Ingram, Stenner, and May 2023; Langridge 2019). This study offered further insight in several ways. Participants placed great value on having a supportive team that embraced uncertainty as a normal part of working life, rather than a sign of weakness or incompetence, and encouraged open and honest discussions among clinicians without fear of embarrassment.

This move towards professional cultures which embrace uncertainty has been reported in the wider healthcare literature (Costa et al. 2022; Hatch 2017; Simpkin and Schwartzstein 2016). It has also been observed that close teamwork and open communication can improve outcomes in the face of clinical complexity and uncertainty (Rosen et al. 2018).

Protected non‐clinical time, for example for case discussions, was seen as particularly important. This has been echoed in research looking at the correlation between lack of non‐clinical time and burnout among FCPs (Nozedar and O’Shea 2023), and in the wider medical literature (Simpkin et al. 2018).

The importance of communication extended to areas such as managing uncertainty by discussing it openly with patients, with participants acknowledging that this approach needs to be individualised. This strategy has produced mixed results in the primary care literature (Cousin, Mast, and Jaunin‐Stalder 2013). While some have suggested that it can lead to stronger clinician‐patient relationships (Armstrong 2018), others have questioned whether sharing uncertainty empowers or frightens patients (Danczak and Lea 2017b).

Participants valued the use of shared decision‐making and the ICE consultation model (Pendleton et al. 2003) to guide these discussions. The ICE model is widely used in healthcare; however, it has been the subject of criticism for its ‘checklist’ approach (BMJ 2016). Shared decision‐making has been noted in the medical literature to have a positive influence on patient satisfaction and outcomes (Barry and Edgman‐Levitan 2012), although it may not always be well received according to participants in this study.

### 5.2 Facilitators and Barriers

Facilitators and barriers to the management of uncertainty are listed in Table 5. These link to the recommendations for practice in Table 6.

**TABLE 5:** Facilitators and barriers.

Facilitators	Barriers
Clinical experience (linked to awareness of when feeling uncertain)	Lack of experience
Wider medical knowledge/training	Complexity of patients/multiple comorbidities
	Lack of training
Use of investigations—for example, imaging, blood tests	Ambiguous findings on investigations
Contextualising findings as incidental/‘normal for age’	Over‐medicalisation—Linked to increasing patient anxiety
Clinical reasoning models to screen for serious pathology	Lack of reliability of traditional physiotherapy assessment techniques/‘Red Flags’
‘Gut instinct’	
Safety netting	Patients not fully understanding safety netting advice or taking it seriously
Flexibility of clinics	Time management—Effect on clinical reasoning
Control over appointment slots for example, for follow ups	Lack of understanding of FCP role—For example, GPs ‘dumping’ complex patients into FCP clinics
Access to colleagues—Primary/secondary care & MSK teams	Lack of access to colleagues—For example, working on different sites or days
Spoke vs. hub model of practice	Spoke vs. hub model of practice
Use of IT systems to facilitate communication links	
Supportive colleagues in primary care and MSK teams	Unsupportive colleagues/fear of being seen as ‘incompetent’
Being able to discuss uncertainty in a ‘safe space’	
Protected non‐clinical time, for example, for complex case discussions	Lack of protected non‐clinical time
Discussing uncertainty with patients	Potential for negative impact on clinician‐patient relationship
Consultation styles—For example, ICE	
Shared decision‐making	Patients wanting to be told ‘what to do’

**TABLE 6:** Recommendations for practice.

Encourage a **supportive team environment** in which uncertainty is seen as a normal part of clinical practice, and is discussed openly and without judgement
Consider the **challenges**—personally and professionally—that FCPs face and what support is needed to maintain job satisfaction, health and wellbeing, and desire to stay in the role
Consider how FCPs might be best placed to adopt a **broader approach** to meet the needs of an ageing population with multiple comorbidities and to support change in health and wellness behaviours, and what additional training they might need to deliver this
Consider what **training** would be most beneficial in supporting FCPs to manage uncertainty—for example, shared decision‐making, challenging conversations, consultation style, time management—and who would be best placed to deliver training, for example, other members of the multidisciplinary team
Consider training/supervision to ensure **consistent** use of physical examination techniques/clusters of tests & **appropriate** use of investigations/explaining investigation results to patients
Consider guaranteed **protected non‐clinical time** to be used for, for example, case discussions, supervision and training
Consider review of **FCP workload and role mix** with a view to reducing burnout
Consider the pros and cons of **hub vs. spoke models** of practice and what allowances/adjustments need to be made to ensure the smooth running of the service/ease of access to colleagues
Consider **flexibility of FCP clinics**, allowing for embargoed follow‐up slots if required

While often seen as facilitators, the use of the ‘traditional’ assessment format and the use of investigations were acknowledged to be ‘double‐edged swords’, often muddying the waters further instead of providing clarity. Many MSK ‘special’ tests, for example, have been widely criticised in the MSK literature for their lack of reliability (Hegedus, Wright, and Cook 2017), while investigations—such as MRI scans for back pain—often show changes that are common in the pain‐free population (Brinjikji et al. 2015). Additional training for FCPs in these areas may need to be implemented—for example, to ensure consistency in assessment and/or consultation techniques and investigation selection, as well as how investigation results are explained to patients.

There was concern that investigations could lead to over‐medicalisation of patients. Reliance on investigations to manage uncertainty, a form of ‘defensive medicine’, is considered to be widespread in medicine, although there is little published evidence of its prevalence within physiotherapy (Finucane et al. 2022). Although often done with good intentions, for example to reassure an anxious patient, it can have detrimental effects on patients, with incidental or age‐related findings being misinterpreted as pathological (Darlow et al. 2017), while even a scan reported as ‘normal’ can increase worry (Van Ravesteijn et al. 2012).

Barriers to successful management of uncertainty include lack of wider medical knowledge and time management. These factors have been previously highlighted as essential skills for FCPs (Langridge 2019); however, there was inconsistency in the training participants received. This may be of relevance to managers of FCP services when considering training needs, particularly in light of recent advice from Health Education England to take advantage of funded FCP courses (HEE 2024).

Time pressures have been strongly linked to uncertainty in primary care (Atkinson, Ajjawi, and Cooling 2011). Most FCP services in the UK adhere to 20‐min appointments (Halls et al. 2020). In this study, even highly experienced practitioners struggled with time management, particularly when seeing more complex patients. This was linked to feelings of burnout and exhaustion, and consideration of leaving the role. Some participants had already reduced their FCP hours due to the stress involved.

### 5.3 Study Limitations and Strengths

It is recognised that the results presented are subjective and influenced by various factors. The researcher's FCP knowledge and background is acknowledged, with steps taken to mitigate and reflect on this as outlined in the Methodology.

Although this is a small study, the sample size achieved the desired theoretical sufficiency and was similar to previous research in this area.

The use of convenience sampling and recruitment via social media may be criticised for only reaching a population that is not representative of FCPs more generally. However, this method did provide participants from different regions of the country with varying levels of experience, and employed by different types of organisation, therefore enhancing the transferability of the study findings. It did not provide ethnic diversity, with eight out of nine participants being ‘White’. In future research, it would be worth considering purposive sampling techniques in conjunction with, for example, the Chartered Society of Physiotherapy's BAME network, to achieve greater ethnic diversity.

Suggestions for future research in this area are given in Table 7.

**TABLE 7:** Suggestions for future research.

How management of uncertainty differs between more experienced vs. less experienced clinicians
Exploration of reasons why FCPs intend to leave role and strategies to address this
Comparing staff retention/intention to leave role between full‐time FCPs and those in a part‐time or split role
Exploration of factors causing burnout among FCPs and strategies to mitigate this
What training would be most appropriate to help FCPs manage uncertainty better
The role of multi‐disciplinary team working to mitigate uncertainty within the FCP role

## 6 Conclusion

This study adds to the FCP literature by providing insight into how FCPs manage uncertainty, and the barriers and facilitators involved. This indicates that the management of uncertainty is a key part of the FCP role, and that uncertainty can provoke a variety of responses from clinicians—positive and negative. The findings of this study may have implications not only for FCPs themselves but also for those involved in the delivery and management of FCP services.

The ability to manage uncertainty was influenced by several factors, including: clinician experience, patient complexity and wider medical knowledge, use of investigations and clinical reasoning models, communication and consultation styles, and access to/working relationships with colleagues. Perhaps the key to successful management of uncertainty was having a supportive team which encouraged open, non‐judgemental discussions about uncertainty.

## Author Contributions

All authors contributed significantly to the study's conception, design, data analysis, manuscript writing, revision, and approval for publication. They are all accountable for ensuring the work's accuracy and integrity and addressing any concerns related to it.

## Conflicts of Interest

The authors declare no conflicts of interest.

## Data Availability

The data that support the findings of this study are available from the corresponding author upon reasonable request.

## Appendix 1 Worked Example of Article Review

Study: Langridge, N. (2019). The skills, knowledge and attributes needed as a first‐contact physiotherapist in musculoskeletal healthcare. *Musculoskeletal Care*, 17:253–260. https://doi.org/10.1002/msc.1401.

### Consolidated Criteria for Reporting Qualitative Studies (COREQ): 32‐Item Checklist

FROM: Tong, A., Sainsbury, P. & Craig, J. (2007). Consolidated criteria for reporting qualitative research (COREQ): a 32‐item checklist for interviews and focus groups. *International Journal for Quality in Health* Care, 19(6):349–357. https://doi.org/10.1093/intqhc/mzm042.

**Table:**

No. item	Guide questions/description	Comment	Reported on page number (or N/A)
**Domain 1: Research team and reflexivity**			
*Personal characteristics*			
1. Interviewer/facilitator	Which author/s conducted the interview or focus group?	The interviews and focus groups were facilitated by the only author, N. Langridge	255
2. Credentials	What were the researcher's credentials? For example, PhD, MD	Not given	N/A
3. Occupation	What was their occupation at the time of the study?	Little information was given except that the researcher was also a clinician working in the same environment as the participants	258
4. Gender	Was the researcher male or female?	Male	253
5. Experience and training	What experience or training did the researcher have?	Not mentioned	N/A
*Relationship with participants*			
6. Relationship established	Was a relationship established prior to study commencement?	It is mentioned that the researcher worked in the same environment as the participants	258
7. Participant knowledge of the interviewer	What did the participants know about the researcher? For example, personal goals and reasons for conducting the research	Not specifically discussed	N/A
8. Interviewer characteristics	What characteristics were reported about the inter viewer/facilitator? For example, bias, assumptions, reasons and interests in the research topic	Not discussed in detail, other than the researcher worked in the same environment as the participants and that this may have influenced data	258
**Domain 2: Study design**			
*Theoretical framework*			
9. Methodological orientation and theory	What methodological orientation was stated to underpin the study? For example, grounded theory, discourse analysis, ethnography, phenomenology, content analysis	The research took an interpretative approach, but other than that little detail is given	254
*Participant selection*			
10. Sampling	How were participants selected? For example, purposive, convenience, consecutive, snowball	Purposive—Participants involved with FCP/APP work. Also included a GP trainer in order to gain a non‐physio perspective	255
11. Method of approach	How were participants approached? For example, face‐to‐face, telephone, mail, email	Managers of MSK services were approached by email and asked to deliver information sheets/consent forms to potential participants. It is unclear how the GP was approached	255
12. Sample size	How many participants were in the study?	Eight for the interviews	255
		Five for the focus group	
13. Non‐participation	How many people refused to participate or dropped out? Reasons?	None	255
*Setting*			
14. Setting of data collection	Where was the data collected? For example, home, clinic, workplace	Interviews—Online (Skype)	255
		Focus group—Not described	N/A
15. Presence of non‐participants	Was anyone else present besides the participants and researchers?	Not mentioned	N/A
16. Description of sample	What are the important characteristics of the sample? For example, demographic data, date	Very basic demographic data given in Table 1 give years of experience as FCP/APP only	255
*Data collection*			
17. Interview guide	Were questions, prompts, and guides provided by the authors? Was it pilot tested?	The interviews were supported by a topic guide developed by the author; however, further details are not given	255
		The focus group was supported by a topic guide that was underpinned by the analysis of the interviews	
18. Repeat interviews	Were repeat interviews carried out? If yes, how many?	No	N/A
19. Audio/visual recording	Did the research use audio or visual recording to collect the data?	Interviews/focus groups were audio recorded only	255
20. Field notes	Were field notes made during and/or after the interview or focus group?	Yes	255
21. Duration	What was the duration of the interviews or focus group?	Interviews lasted approximately 35–40 min. Focus group—No details given	255
22. Data saturation	Was data saturation discussed?	No	N/A
23. Transcripts returned	Were transcripts returned to participants for comment and/or correction?	Not mentioned	N/A
**Domain 3: Analysis and** **findings**			
*Data analysis*			
24. Number of data coders	How many data coders coded the data?	Two	255
25. Description of the coding tree	Did the authors provide a description of the coding tree?	No	N/A
26. Derivation of themes	Were themes identified in advance or derived from the data?	Derived from data	255
27. Software	What software, if applicable, was used to manage the data?	N/A	N/A
28. Participant checking	Did participants provide feedback on the findings?	Not mentioned	N/A
*Reporting*			
29. Quotations presented	Were participant quotations presented to illustrate the themes/findings? Was each quotation identified? For example, participant number	Yes to both—Although no details were given on individual participants in relation to the participant number	256–257
30. Data and findings consistent	Was there consistency between the data presented and the findings?	Yes	256–257
31. Clarity of major themes	Were major themes clearly presented in the findings?	Yes	256–257
32. Clarity of minor themes	Is there a description of diverse cases or discussion of minor themes?	No	N/A

## Appendix 2 Interview Guide

**Introduction**: Thank you for taking part in this project. I am interested in understanding your perspectives on how you manage uncertainty as part of your FCP role. As an MSK FCP, your perspective will be invaluable. There are no right or wrong answers. I am very interested in your thoughts and opinions, whether they are positive, negative, or neutral. Please feel free to pause the interview if you need to take a break, or let me know if you need to end the interview for any reason. Also, just to be clear that you don't have to answer every question—just let me know if you want to move on to the next question. Before we start the interview, do I have your permission to record this session?

**1. Opener**: Please start by telling me about your FCP role.

*Prompts:* How long have you been working as an FCP? How many days a week do you work as an FCP? Do you have a split role?

**2. Can you think of a time when you experienced uncertainty related to your FCP role?** Tell me what happened.

*Prompts:* For example, a situation related to: uncertainty around diagnosis, missing serious pathology, boundaries of the role, when to refer on… Or related to the patient, for example: managing patients' expectations in relation to the evidence; patients' personal/social contexts or their emotions/mental health.

**3. Can you tell me about how you managed that situation?**

*Prompts:* Did you discuss it with a colleague? Did you consider further investigations? Did you refer to any evidence/guidelines?

*Follow up questions*: Did you find this strategy helpful? Were there any negative aspects to this strategy?

**4. Can you think any other occasions when you experienced uncertainty related to your FCP role?**

*Prompts:* as per Q2.

**5. Are there any particular aspects of the FCP role that make you feel uncertain?**

*Follow up question:* What is it about these aspects of the role that makes you feel uncertain?

**6. Is there anything related to your role that makes management of uncertainty easier?**

*Prompts:* For example, getting support from others—supervision (formal or informal)/talking to primary care colleagues, for example GPs; training; consulting guidelines/research.

*Follow up question:* What do you find most helpful?

**7. Can you think of anything that makes management of uncertainty more difficult?**

*Prompts:* For example, lack of supervision; being isolated from colleagues/other members of the primary care team, lack of training, time management.

*Follow up question:* What do you find most challenging?

**8. Do you ever discuss your uncertainty with your patients?**

*Follow up questions:* Do you find this a helpful strategy? Has it ever had any negative consequences?

**9. Do you think that managing uncertainty in your role has an impact on you—positive or negative?** Can you expand further?

*Follow up questions:* Do you find it exciting/stimulating? Has it caused you to experience stress/burnout? Has it made you think about whether you want to remain in the role?

**10. Is there anything else you would like to add that we haven't covered today?**

**Closing statement:** Thank you for valuable time and insights, I greatly appreciate it. The next stage is, I will transcribe the interview. Would you like a copy of the transcription to check its accuracy?

## Appendix 3 Table of Themes & Codes

**Table:**

Theme/subtheme	Theme/subtheme synopsis	Examples of codes
**Theme 1**: Being comfortable to being uncomfortable *Subtheme: Barriers & facilitators to being comfortable with uncertainty*	Uncertainty is prevalent in primary care; the first step to managing it is acknowledging its present and being comfortable with uncertainty. Clinical experience was identified as a key factor in managing uncertainty although it could also make participants worry about how much they still didn't know. Participants identified several barriers and facilitators related to the management of uncertainty. These included: Wider medical knowledge and training; use of investigations; safety netting; complexity of patients; strategies for screening for serious pathology; time management and flexibility of clinics	Uncertainty is pervasive in FCP; being comfortable with uncertainty; importance of recognising when you are uncertain; OK not to have all the answers; clinical experience is essential for managing uncertainty; clinical experience trumps other skills/courses/roadmap to practice; clinical experience linked to patient mileage—Knowing what's ‘normal’; lack of experience may be a barrier; concern around how less experienced clinicians deal with uncertainty; experience as a ‘double‐edged sword’; uncertainty over missing something serious; use of investigations to mitigate; use of traditional physiotherapy examination to mitigate; awareness that investigations/tests not always reliable; test findings ambiguous; fear of over‐medicalising patients; gut instinct; anxious patients make us more anxious; complexity of patients; diagnostic uncertainty; MSK vs. non‐MSK cause of symptoms; safety netting; time management—Effect on clinical reasoning; misunderstanding of FCP role by primary care colleagues; having control over clinics
**Theme 2**: Team work makes the dream work *Subtheme: Making uncertainty normal*	This theme examines the importance of having a supportive team and good working relationships with colleagues in primary and secondary care, as well as with MSK teams. Good access to the primary care multi‐disciplinary team was seen as essential, and the use of IT systems was integral to this. Participants put great value on having a supportive MSK team which encouraged open discussions on uncertainty. Participants felt that uncertainty should be seen as a normal part of working life rather than a sign of ‘failure’. Lack of support from colleagues and lack of protected non‐clinical team were seen as barriers to the management of uncertainty	Access to GP; access to the wider primary care team; lack of access to GP; good working relationships; importance of MDT working; involving MDT in training; access to secondary care colleagues; use of IT to facilitate communication; importance of protected non‐clinical time; lack of non‐clinical time; value of discussions with MSK colleagues; ease of access to MSK colleagues; lack of access to MSK colleagues; isolation; supportive team environment/safe space; healthy working culture—Open to discussing uncertainty
**Theme 3**: Navigating uncertainty with patients	Having honest and open discussions with patients about uncertainty was seen by participants as a valuable tool in the management of uncertainty; however, it was not always appreciated by patients wanting more direct answers or wanting to be told what to do. The use of consultation styles (e.g., based on the ICE model) and shared decision‐making with patients were seen as facilitators for having discussions about uncertainty with patients	Discussing uncertainty with patients; being honest/open with patients about uncertainty; making patients feel you are listening; making patients feel they are being taken seriously; shared decision‐making with patients; positive responses to discussing uncertainty; negative responses to discussing uncertainty; tailoring discussions to individual patients; use of ICE model of consultation
